# Transforming Food Biowaste into Selective and Reusable Adsorbents for Pesticide Removal from Water

**DOI:** 10.3390/ma17225499

**Published:** 2024-11-11

**Authors:** Vedran Milanković, Tamara Tasić, Snežana Brković, Nebojša Potkonjak, Christoph Unterweger, Igor A. Pašti, Tamara Lazarević-Pašti

**Affiliations:** 1 Vinča Institute of Nuclear Sciences—National Institute of the Republic of Serbia, University of Belgrade, Mike Petrovica Alasa 12–14, 11000 Belgrade, Serbia; tamara.tasic@vin.bg.ac.rs (T.T.); snezana.miulovic@vin.bg.ac.rs (S.B.); npotkonjak@vin.bg.ac.rs (N.P.); tamara@vin.bg.ac.rs (T.L.-P.); 2 Wood K Plus—Kompetenzzentrum Holz GmbH, Altenberger Strasse 69, 4040 Linz, Austria; c.unterweger@wood-kplus.at; 3 Faculty of Physical Chemistry, University of Belgrade, Studentski Trg 12–16, 11158 Belgrade, Serbia; igor@ffh.bg.ac.rs

**Keywords:** spent coffee grounds, waste-to-value, activated carbon materials, chlorpyrifos, malathion, adsorption

## Abstract

With growing concerns regarding environmental pollution and the need for sustainable waste management practices, this study investigates the potential of utilizing spent coffee grounds (SCG) as a precursor for producing functional carbon materials aimed at organophosphorus pesticide remediation under environmentally relevant conditions. Carbonization of SCG is followed by various activation methods, including treatment with potassium hydroxide, phosphoric acid, and carbon dioxide, individually or in combination. The resulting biochars are systematically analyzed for their adsorption performance towards malathion and chlorpyrifos. Screening tests revealed a selective adsorption preference towards aromatic chlorpyrifos over aliphatic malathion. Activation processes significantly influence adsorption kinetics and efficiency, with physical activation showing notable adsorption rates and capacity enhancements. Moreover, the SCG-derived biochars exhibit a pronounced dependency on adsorption temperature. Adsorption, regeneration, and reuse of the most promising material are tested in a real, spiked tap water sample, proving that the presence of ions in tap water did not affect the adsorption and that the material has the potential to be reused more than ten times. This work proposes a straightforward approach for recycling SCG by converting it into functional carbon materials, underscoring the importance of selecting the appropriate activation processes and conditions for practical applications in pesticide remediation.

## 1. Introduction

Biowaste poses a significant challenge globally, driven by its environmental, health, and economic impacts [1]. It comprises organic waste material from food processing, preparation, and consumption, including agricultural by-products and kitchen scraps. Food biowaste is a significant contributor to this problem. As biowaste decomposes, it releases greenhouse gases like CO_2_, impacting climate change. Economically, food biowaste represents a loss of valuable food resources, highlighting inefficiencies in resource use that could otherwise support broader societal needs [2,3].

Effectively addressing biowaste involves innovative recycling technologies, such as converting biowaste into high-value products. Thermochemical conversion processes can transform biowaste into activated carbon and biochar [4], and their additional chemical, physical, and physicochemical activation can enhance surface area and porosity, making them suitable for a range of environmental applications, including water purification [5]. The development of selective adsorbents is highly demanding and time consuming and requires many resources. The right choice of activation method allows the tailoring of the biowaste material to better suit adsorption requirements for specific contaminants, enabling the preparation of low-cost and eco-friendly adsorbents [6].

By subjecting the biowaste to activating agents or specific conditions, activation results in creating a more intricate porous structure and an expanded surface area. This process significantly impacts the materials in several key ways. Firstly, activation promotes the formation of micropores and mesopores, providing additional sites for interactions with target compounds [7]. Secondly, the activation process substantially increases the material’s surface area, facilitating improved adsorption capacity by accommodating more molecules and enhancing efficiency in capturing and immobilizing contaminants [8,9].

Among the various forms of food biowaste, spent coffee grounds (SCG) present a unique challenge due to their volume and treatment difficulties. Annually, the world produces approximately 10.5 million metric tons of coffee, primarily in countries like Brazil and Vietnam, leading to substantial quantities of SCG [10]. If not managed properly, these by-products can cause severe environmental issues, including air, water, and land pollution. The composting process relies on oxygen (O_2_) to decompose organic materials and generates emissions like carbon dioxide (CO_2_), methane (CH_4_), nitrous oxide (N_2_O), ammonia (NH_3_), and various biogenic volatile compounds. Methane (CH_4_) and nitrous oxide (N_2_O) are especially significant, as they are potent greenhouse gases with a strong influence on global warming potential. The release of these gasses from SCG contributes to smog, ozone formation, and climate change, underscoring the need for their proper management [11,12]. Therefore, SCG represent a substantial biomass waste stream, offering a promising path for sustainable resource utilization. In this context, transforming SCG into biochar presents an eco-friendly and cost-effective solution with diverse applications, particularly in environmental remediation [13,14].

**Scheme 1:**
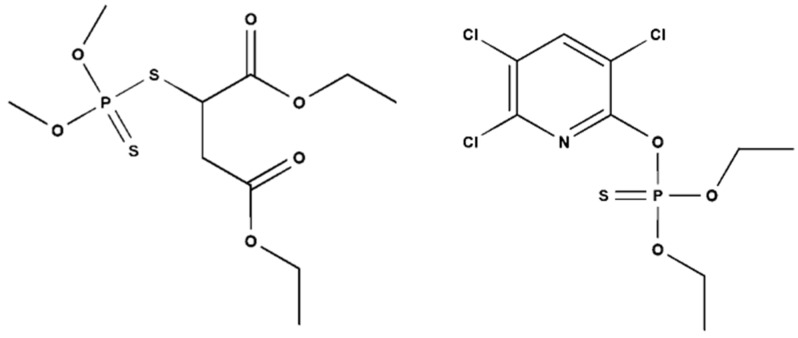
Malathion (**left**) and chlorpyrifos (**right**) chemical structures.

Organophosphorus pesticides (OPs), widely employed in agriculture, pose significant environmental concerns due to their persistence and potential adverse effects on ecosystems and human health [15,16]. In particular, malathion (MLT) and chlorpyrifos (CHP) have long been integral components of agricultural practices for their efficacy in pest control (structures shown in Scheme 1). MLT, an organophosphate insecticide, is widely utilized in agriculture and public health programs [17]. Similarly, CHP, a broad-spectrum organophosphate, finds extensive application in the protection of various crops [18]. However, the unselective use of these pesticides has raised environmental concerns, with documented contamination in soil, water, and food sources [19,20,21]. Both MLT and CHP are known to be toxic to non-target organisms, including aquatic life and beneficial insects, posing a significant threat to ecosystem integrity [22,23].

Recognizing the urgent need for sustainable solutions to mitigate the adverse effects of these pesticides, it is essential to investigate their remediation. Various strategies have been employed to remediate OPs. Among the biological approaches, microbial degradation has shown promise, harnessing the enzymatic capabilities of certain microorganisms to break down OP compounds into less harmful by-products [24]. Phytoremediation, utilizing plants with the ability to accumulate and metabolize pesticides, offers a green and sustainable strategy for OP removal from soil and water [25]. Chemical remediation methods involve oxidation and reduction reactions, where adding oxidizing or reducing agents transforms OP pesticides into less toxic forms. Advanced oxidation processes, such as photocatalysis and ozonation, deploy reactive species to degrade OPs [26]. Adsorption, particularly if employing materials like activated carbon and biochar, is highly efficient for capturing and immobilizing OP compounds from soil and water matrices. The porous structure of these adsorbents provides a large surface area and numerous binding sites, enhancing the capacity to attract and retain OP pesticides [27]. Additionally, adsorption is a versatile and environmentally friendly process, avoiding the need for complex chemical reactions or the generation of harmful by-products. With its well-established adsorption capabilities, activated carbon has been widely employed in water treatment and soil remediation [28].

This study focused on biochar production from SCG by carbonization at 650 °C, the lowest temperature at which all SCG organics are combusted [28]. We investigated how various activation methods impact the adsorption capacity of SCG for CHP and MLT under environmentally relevant conditions [29,30,31], as well as their regeneration and reuse.

The novelty of this work lies in its sustainable approach, using SCG as a precursor for biochar production, coupled with a comprehensive analysis of various activation methods (chemical, physical, and combined). The study uniquely explored the selective adsorption of organophosphorus pesticide aromatic CHP over aliphatic MLT under environmentally relevant conditions. The adsorption, regeneration, and reuse of the most promising material were tested in a real, spiked water sample. A detailed characterization and comparison with other biomass-derived adsorbents underscored the superior efficacy of SCG biochar, making it a promising and practical solution for pesticide remediation in real-world scenarios.

This research paper supports and advances the UN’s sustainable development goals, in particular SDG 6 (Clean Water and Sanitation) by remediation of OPs, SDG 12 (Responsible Consumption and Production), and SDG 13 (Climate Action) by using climate-harmful biowaste (SCG) in the production of carbon materials [32].

## 2. Materials and Methods

### 2.1. Preparation of Carbon Materials

Materials were synthesized from SCG through distinct processing methods. Table 1 presents conditions for the synthesis of the materials with corresponding yields, which are given in the Supplementary Materials section.

Coffee (purchased from the local market (80% Arabica and 20% Robusta)) was brewed (treated with boiling water) and left for 2 h at room temperature until it cooled. Next, the coffee grounds were separated with filtration and left to dry at room temperature for 24 h. Then, in order to release the leftover moisture, the obtained spent coffee grounds were dried in the oven at 80 °C for 1 h. All materials were thermally treated at 650 °C, with a heat rate of 5 °C min^−1^ in N_2_ atmosphere. SCG–650 underwent thermal treatment at 650 °C without additional activation, representing the baseline material. Chemical activation using either KOH or H_3_PO_4_ (c = 1 mol dm^−3^; weight ratio = 2:1 (material:activator solution)) was introduced to SCG–650K and SCG–650P, respectively. SCG–650C was prepared by thermochemical conversion of SCG at 650 °C, with a heat rate 5 °C min^−1^ in N_2_ atmosphere and additionally physically activated in an isothermal step using CO_2_ flow 100 L h^−1^ for 1 h. The combined activation method in SCG–650KC and SCG–650PC involved initial chemical activation (KOH or H_3_PO_4_) of SCG–650 followed by CO_2_ physical activation in the isothermal step (1 h). All materials were ground for 15 min using a pestle and mortar after each thermal treatment step. Afterward, the materials were washed step-wise using, successively, 50 mL NaOH (0.1 mol dm^−3^), 50 mL HCl (0.1 mol dm^−3^), and 50 mL deionized water and finally suspended in 50 mL 50 vol.% ethanol to obtain stock suspension (2 mg mL^−1^).

### 2.2. Materials’ Characterization

ATR-FTIR spectra were acquired using a Nicolet iS20 FT-IR spectrophotometer from Thermo Fisher Scientific, Waltham, MA, USA. The spectral data were collected within a wavenumber range of 4000 to 500 cm^−1^, employing 64 scans and a resolution of 4 cm^−1^. The spectra of adsorbents were recorded prior to and after the adsorption of OP. A PhenomProX scanning electron microscope (SEM) from Thermo Fisher Scientific, Waltham, MA, USA, was utilized to examine sample morphology and elemental composition, incorporating energy-dispersive X-ray analysis (EDX). Zeta potential (ZP) measurements were conducted utilizing a Nano ZS zetasizer system equipped with a 633 nm He-Ne laser, Malvern Instruments, Malvern, UK. The specific surface area and total pore volume of the materials were measured using an Autosorb-iQ gas sorption system (Anton Paar QuantaTec Inc., Graz, Austria). Nitrogen adsorption was performed at a constant temperature of −196.15 °C. Before the analysis, the samples underwent outgassing for 5 h at 300 °C under vacuum conditions to eliminate any adsorbed impurities. This procedure provided a thorough evaluation of the materials’ textural characteristics.

### 2.3. Adsorption Experiments

In the experimental procedure, a mixture comprising 0.5 mL of SCG–650 materials stock dispersion (2 mg mL^−1^, pH = 6) and 0.5 mL of specified concentrations of OPs (Pestanal, Sigma Aldrich, Søborg, Denmark) stock solutions (prepared in 50 vol.% ethanol in water) were prepared to achieve the desired concentration of both the adsorbents and OPs. A pH value of 6 was chosen, as the pH value of most contaminated surface waters is around that value [33]. The mixtures were put on a laboratory shaker for the designated duration. Subsequently, centrifugation at 14,500 rpm was applied, and the supernatant was filtered through a nylon membrane. For the quantification of OP concentrations, ultra-performance liquid chromatography (UPLC) analysis was carried out using the Waters ACQUITY UPLC system (Waters GmbH, Eschborn, Germany) and a photodiode array (PDA) detector managed by Empower 3 software.

An ACQUITY UPLC™ BEH C18 column (1.7 μm, 100 mm × 2.1 mm) was employed under isocratic conditions with 10% acetonitrile (J.T. Baker, Phillipsburg, NJ, USA) in water (*v*/*v*) as mobile phase A and pure acetonitrile as mobile phase B. In all cases, the eluent flow rate was maintained at 0.2 mL min^−1^, with an injection volume of 5 μL. Mobile phase composition for CHP was 20% A and 80% B, and for MLT, it was 40% A and 60% B. The retention times were 2.6 min for MLT and 2.7 min for CHP. Detection of both OPs was performed at 200 nm. Control experiments were conducted identically but without the presence of an adsorbent.

Adsorption experiments were performed under environmentally relevant conditions (temperatures of 25, 30, and 35 °C; pH = 6; and pesticide concentrations ranging from 5 × 10^−6^ to 5 × 10^−4^ mol dm^−3^ [29,30,31]) to examine the potential of SCG–650 materials’ application in the real world. All experiments were performed in triplicate, and the mean values are shown on graphs. The highest deviation of the mean value is presented as the error bar.

The adsorption kinetics was investigated by incubating 1 mg mL^−1^ of materials with CHP (5 × 10^−5^ mol dm^−3^) for various time intervals (ranging from 1 min to 1440 min) at 25 °C. The concentrations of CHP remaining in the solution at given moments of time were measured using UPLC. The adsorbed amounts of CHP were obtained by subtracting the amounts of CHP remaining in the solution from the initial amount present in the solution. The experimental data were analyzed using non-linear pseudo-first-order (PFO), pseudo-second-order (PSO), Elovich, and intraparticle diffusion (IPD) kinetic models [34].

To obtain experimental data for isotherm studies of pesticide adsorption onto these materials, 1 mg mL^−1^ of material was incubated for 1440 min with CHP in the concentration range from 5 × 10^−6^ to 5 × 10^−4^ mol dm^−3^ at 25 °C, 30 °C, and 35 °C. The experimental data were analyzed using non-linear Freundlich, Langmuir, Temkin, and Dubinin–Radushkevich isotherm models. Equations of all used models are given in Table S1 (Supplementary Materials).

The parameters R^2^ and χ^2^ were calculated to evaluate how well the experimental data fit the selected adsorption models. R^2^, the coefficient of determination, measures the proportion of variance in the observed data explained by the model; values close to 1 indicate that the model accurately represents the data. Generally, a value of R^2^ above 0.9 is considered indicative of a strong fit, although this threshold can vary depending on the study and model complexity. The χ^2^ parameter, often used in adsorption studies, assesses the deviation between predicted and observed values, offering insight into the model’s predictive power. A lower χ^2^ value indicates smaller residuals, suggesting a better fit. Combined, high R^2^ values and low χ^2^ values generally confirm that the chosen model accurately reflects the interaction mechanisms between the adsorbent and the adsorbate, helping to validate the appropriateness of the model in describing adsorption behavior in the system under study.

## 3. Results and Discussion

### 3.1. Physicochemical Characteristics of the Materials

**Figure 1:**
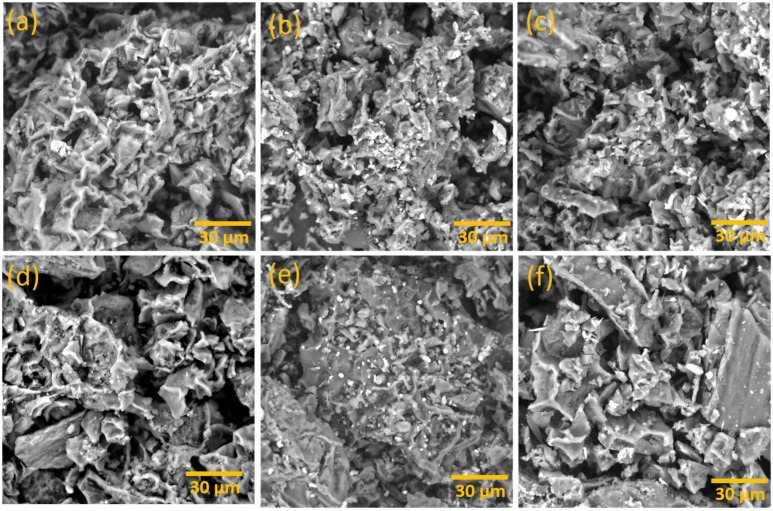
SEM micrographs (magnification ×2000) of: (**a**) SCG–650, (**b**) SCG–650K, (**c**) SCG–650P, (**d**) SCG–650C, (**e**) SCG–650KC, (**f**) SCG–650PC.

The SEM micrographs (Figure 1) display the morphology of the materials, which remained unchanged during the activation process, maintaining a porous and spongy-like structure with unevenly distributed pores consistent with the original morphology of raw SCG [35].

The elemental compositions of investigated materials (Table 2) exhibited distinct atomic percentages of various elements in carbon materials derived from SCG. It clearly suggested that the activation method impacted the chemical composition of the material’s surface.

Carbon, oxygen, and nitrogen were present in significant proportions, indicating the organic nature of the precursor, with carbon being the predominant component in all materials. Notably, the atomic percentage of oxygen increased with the activation process, particularly in materials activated with KOH, indicating a primary influence on surface oxygen functional groups.

As anticipated, materials activated with KOH and H_3_PO_4_ showed the presence of potassium and phosphorus, respectively. Minor elements such as magnesium, calcium, sodium, chlorine, and sulfur contributed to the elemental profile in varying but relatively small amounts. These findings suggest that different activation processes can be employed to tailor the material’s surface for specific applications.

**Figure 2:**
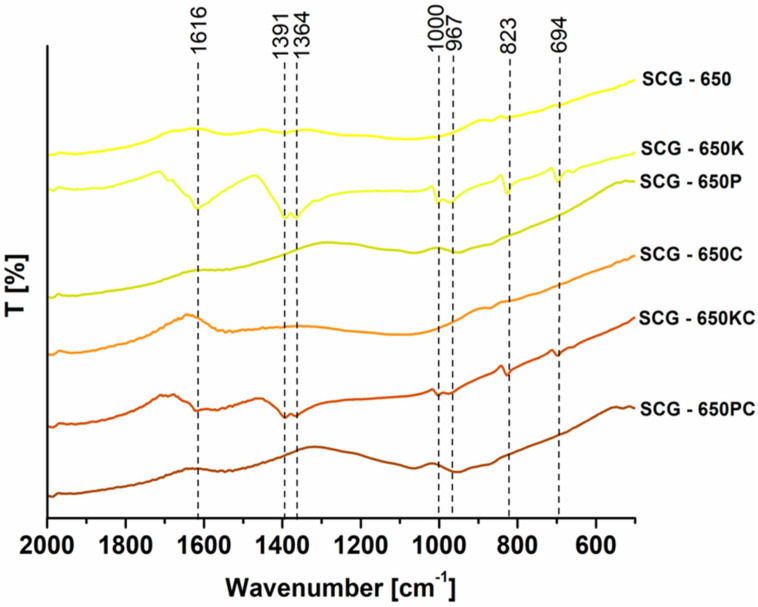
ATR-FTIR spectra of SCG–650 materials.

The ATR-FTIR spectra of all materials are shown in Figure 2. It can be seen that, by the temperature of 650 °C, the SCGs remained almost without functional groups. Also, the activation process with CO_2_ and H_3_PO_4_ did not introduce additional functional groups. Meanwhile, the activation with KOH led to several changes in the ATR-FTIR spectrum. The band at 1616 cm^−1^ was attributed to the stretching of the C=C in the aromatic rings, while the bands at 1391 cm^−1^ and 1364 cm^−1^ were attributed to C-H in-plane deformation vibrations [36]. The bands at 967 cm^−1^ and 1000 cm^−1^ signified the C-O bending vibrations [37,38], and the C-H bending vibrations in the aromatic ring can be seen at 823 cm^−1^ and 694 cm^−1^ [39].

**Figure 3:**
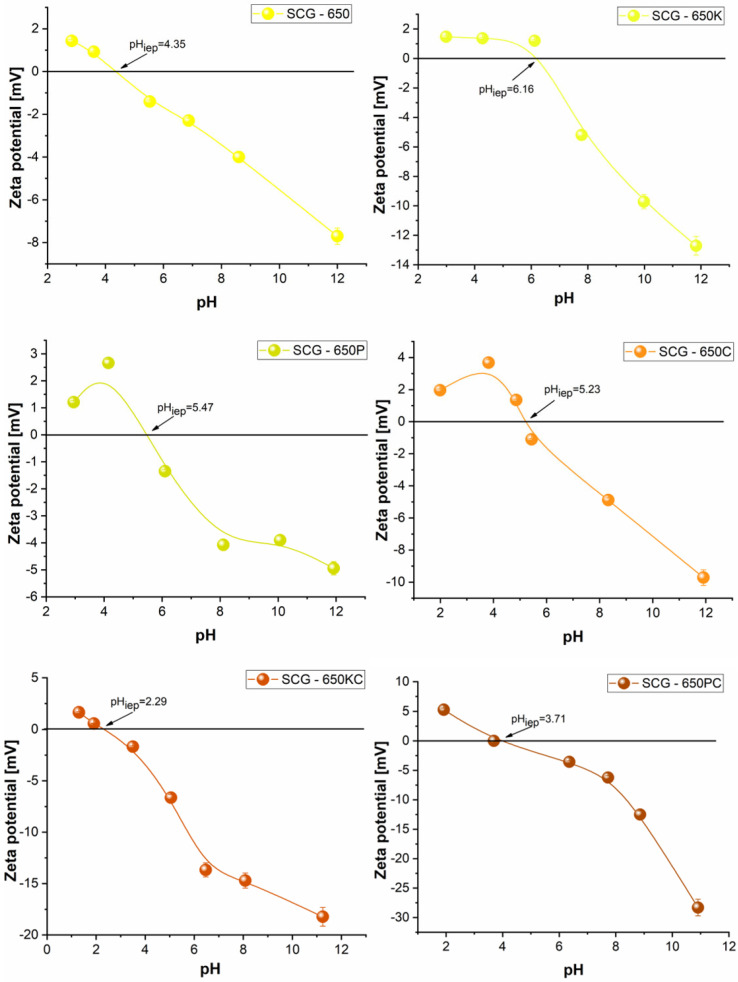
Dependence of zeta potential of pH for all investigated materials.

Zeta potential measurements were conducted to assess the charge of the materials and determine their isoelectric points. Specifically, 1 mL of a 0.5 mg mL^−1^ concentration of the materials was titrated using HCl and NaOH to cover a pH range from 1 to 14 (Figure 3). The isoelectric points for SCG–650, SCG–650K, SCG–650P, SCG–650C, SCG–650KC, and SCG–650PC were found to be 4.35, 6.16, 5.47, 5.23, 2.29, and 3.71, respectively. Given that the initial pH value of all materials suspended in 50% ethanol was 6, it can be concluded that all materials, except SCG–650K, exhibited a negatively charged surface under the conditions employed for adsorption measurements.

The results of the BET measurements and total pore volume determination, as summarized in Table 3, revealed that all the samples had relatively small specific surface areas (S_BET_) and total pore volumes (V_tot_). After the chemical activation of material SCG–650, the specific surface area and total pore volume highly increased, while in the case of physical activation, S_BET_ increased but V_tot_ decreased, indicating the formation of a larger number of smaller pores on the surface of the material. Combined activation KOH/CO_2_ showed multiple times higher values of these parameters, but H_3_PO_4_/CO_2_ impacted their decrease, implying that the results obtained by combining the activation methods could not be linked to the results obtained for the materials with separate activations.

The significant increase in specific surface area observed with combined KOH/CO_2_ activation can likely be attributed to the synergistic effects of these two agents on the biochar structure. KOH activation, known for enhancing porosity, particularly promoted micropore formation by etching the carbon matrix, removing organic components, and creating new pore structures. Meanwhile, CO_2_ activation further developed these micropores and simultaneously broadened them into mesopores by promoting additional carbon gasification. The combination of these two activation agents maximized pore development, as KOH first generated extensive micropores and weakened the carbon structure, making it more susceptible to CO_2_ activation. CO_2_ could then more effectively act on this altered matrix, further expanding the pore network, which resulted in a substantial increase in the specific surface area.

On the other hand, the combined activation using H_3_PO_4_ and CO_2_ likely led to a decrease in the specific surface area due to competing mechanisms that reduced effective pore formation. Phosphoric acid (H_3_PO_4_) generally acted as a crosslinking agent within the carbon matrix, which could promote some initial porosity but also limit the extent of further pore expansion by creating stable phosphate ester linkages. These linkages can hinder the action of CO_2_, preventing it from further expanding or forming new pores in the carbon structure. When H_3_PO_4_ and CO_2_ were used together, the crosslinked network induced by H_3_PO_4_ activation likely reduced the extent of carbon gasification that CO_2_ would otherwise facilitate, leading to a lower specific surface area. This effect contrasted sharply with the KOH/CO_2_ combination, where KOH structurally weakened the matrix, allowing CO_2_ to increase porosity. The H_3_PO_4_/CO_2_ combination, on the other hand, stabilized the structure in a way that resisted pore development, showing that activation effects were not simply additive but rather depended on the interaction between specific agents and the carbon matrix.

### 3.2. Adsorption Experiments

#### 3.2.1. Screening Test

At the beginning, a screening test was performed, which involved determining the percentage of adsorption of MLT and CHP with an initial concentration of 5 × 10^−5^ mol·dm^−3^ on all the synthesized material within 1 h. The results of the screening test are shown in Table 4.

The screening test revealed that chemically activated materials had a lower affinity for MLT adsorption than the carbonized material SCG–650. Therefore, they were not further analyzed, and the material SCG–650K was not used for CHP adsorption for the same reason. In addition to this, SCG–650K exhibited a positive surface charge, indicating weak repulsion between CHP and this material [40]. The minimal adsorption of MLT by all investigated materials (below 2%), including SCG–650KC, which had the highest specific surface area and pore volume, indicated that the surface area alone did not dictate MLT adsorption efficiency in this case. Regardless of the specific surface area, the consistently low adsorption across all materials suggested that other structural factors (likely surface chemistry or molecular orientation) were more critical for interaction with MLT. Specifically, the surface characteristics and molecular configuration of SCG-derived biochars may not have favored interactions with MLT. Unlike aromatic compounds (like CHP), which are generally more effectively adsorbed due to π-π interactions with biochar’s carbon matrix, MLT lacks the aromaticity needed for such favorable interactions. This limitation means that, even with an increased surface area, there was no significant enhancement in MLT adsorption. The shape of the pores could also influence the selectivity of the adsorbent. Even though the percentage of adsorbed MLT increased with physical activation and a combination of KOH and physical activation, the percentage was low under the given conditions, so it is considered that the application of these materials for its adsorption would be non-economical. The screening test indicated that the chemical activation of SCG–650 with both KOH and H_3_PO_4_ negatively impacted the adsorption of CHP. In contrast, the physical activation with CO_2_ and combined activation with H_3_PO_4_ and CO_2_ amplified the adsorption efficiency. So, this work further focused on investigating the kinetics, isotherms, and thermodynamic parameters of CHP adsorption onto synthesized materials, as well as determining the reasons for the selective behavior of these materials.

All stated above imply that SCG-derived materials, carbonized at 650 °C, are highly selective regarding the structure of the adsorbate, as they adsorb aromatic CHP in a much greater percentage compared to aliphatic MLT. The absence of changes in the FTIR spectra of the adsorbent before and after the adsorption (Figure S1, Supplementary Material) indicated that the mechanism of the adsorption was physisorption. Taking this into account, it can be deduced that the probable mechanism of the CHP adsorption onto these materials was the π-π stacking of the aromatic moiety of this OP and aromatic moieties. Also, as S_BET_ and V_tot_ were low and the materials almost did not adsorb MLT, it can be concluded that very small amounts of MLT can enter these pores, probably due to steric hindrance.

#### 3.2.2. Adsorption Kinetics

**Figure 4:**
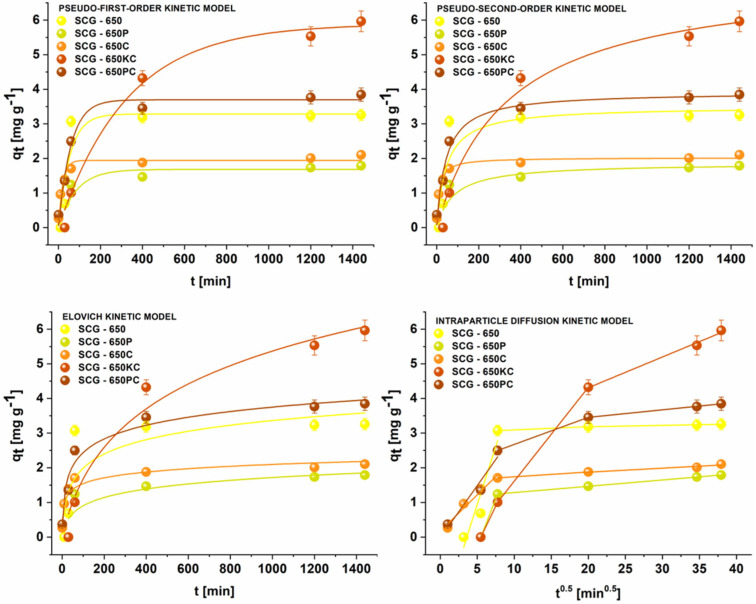
Graphical representations of experimental results fitting with kinetic models.

The adsorption kinetic experiments were performed as described in Section 2.3, and the corresponding graphs are given in Figure 4, while the obtained kinetic parameters with corresponding R^2^ and χ^2^ values are provided in Table 5.

The graphical representation indicates that the equilibrium between CHP and the carbon materials was achieved after 400 min, except for SCG–650KC, where the equilibrium was reached after 1440 min. For consistency, all further experiments were performed at a 1440 min incubation time.

The obtained parameters showed the difference in the kinetic behavior of the materials due to the different activation processes applied. The experimental data for the adsorption onto SCG–650 and SCG–650P did not fit well according to R^2^ in PFO and PSO but it was slightly better for PFO. Experimental data for the other materials fit well into both PFO and PSO. According to the q_e_ values that were similar for both PFO and PSO, it can be seen that the SCG–650KC had the highest adsorption efficiency for the given conditions. The adsorption process was considered slow as the equilibrium was reached after 24 h. The parameters of the Elovich kinetic model confirmed the previously stated, as the initial rate was lower than the desorption constant of the adsorption.

The IPD kinetic model plot displays two linear stages for the CHP adsorption onto all materials, except for adsorption onto SCG–650KC and SCG–650PC, where the adsorption process had three stages. The decline in k_id_ values across stages corresponded to the gradual slowing down of the adsorption process as the boundary layer value increased through the stages, signifying an increase in the molecules adsorbed on the material’s surface. Ultimately, the boundary layer reached a value similar to the q_e_ values obtained from PFO and PSO, and k_id_ reached very low values, signifying the completion of the adsorption process.

#### 3.2.3. Adsorption Isotherm Studies

**Figure 5:**
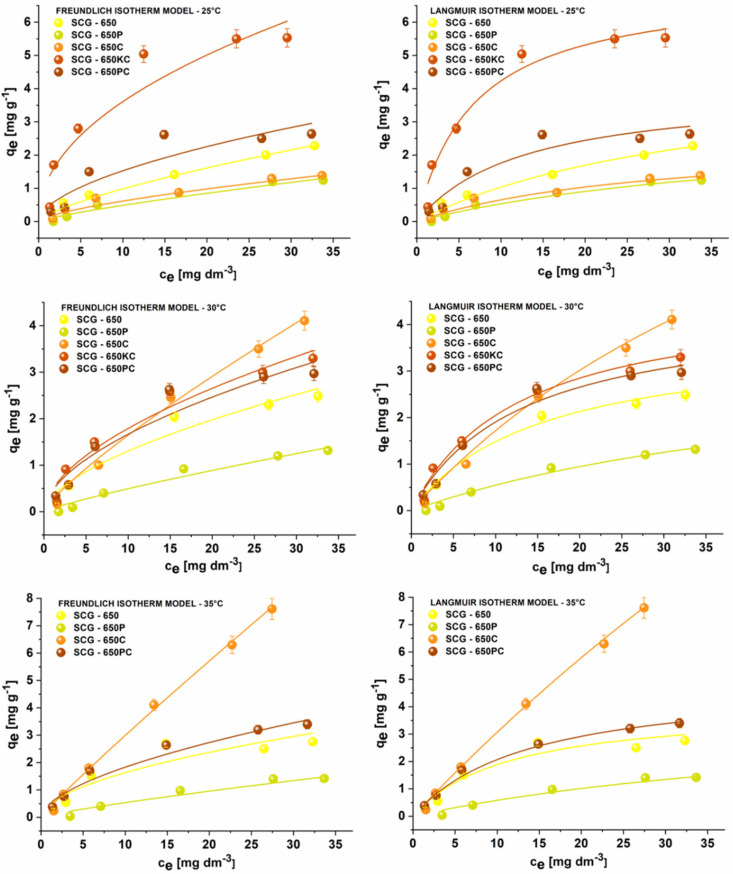
Graphical representation of Freundlich and Langmuir isotherm models for CHP adsorption onto all investigated materials at 25, 30, and 35 °C.

The adsorption isotherm experiments were performed as described in Section 2.3. The corresponding graphical representations are given in Figure 5 (Freundlich and Langmuir), while the obtained parameters are provided in Table 6. The graphical representations of the Temkin and Dubinin–Radushkevich isotherm models (Figure S2) and the tables containing corresponding R^2^ and χ^2^ values (Tables S2–S4) can be found in Supplementary Materials.

First, it can be noticed that the SCG–650KC material did not adsorb CHP at 35 °C under the given experimental conditions, signifying the importance of the adsorption temperature.

By observing the parameters (Table 6), it can be seen that both the Freundlich and Langmuir isotherm models described the adsorption process of CHP onto all the materials at all investigated temperatures well. It suggests that the material’s surface was energetically not entirely homogenous, as could be expected due to the used precursor and the activation processes. At all adsorption temperatures, the n parameter from the Freundlich isotherm model was greater than 1 for all materials, indicating the favorability of the processes. However, with the increase in the adsorption temperature, the n value decreased in all cases, except for the adsorption of CHP onto SCG–650, where it increased.

The maximum amount of the CHP that could be adsorbed by 1 g of material obtained from the Langmuir isotherm model at 25 °C was in the range from 2.5 to 7.1 mg, at 30 °C it was in the range from 3.8 to 12.3 mg, and at 35 °C, it was in the range from 3.9 to 51.7 mg. SCG–650KC showed the best adsorption properties for the removal of CHP at 25 °C, while at higher temperatures, SCG–650C had the best efficiency. With increasing the adsorption temperature, the q_max_ values increased for the adsorption of CHP onto SCG–650P, SCG–650C, and SCG–650PC, while in the other cases, it decreased.

From the above presented, it was obvious that some SCG-derived materials showed improved adsorption performance with temperature, while others exhibited a reduced capacity or efficiency, indicating that these biochars were not universally optimized for all thermal conditions. Tailoring these biochars for particular temperature ranges, perhaps through controlled activation or surface modifications, could enhance their suitability for targeted adsorption applications. This indicates that selecting the appropriate thermal treatments and activation processes is crucial to achieving optimal adsorption efficiency across various environmental conditions.

The parameter b_T_ obtained from the Temkin isotherm model, representing the adsorption heat, showed an increase with the adsorption temperature, except in the case of CHP adsorption onto SCG–650KC. It led us to the assumption that the adsorption processes were endothermic in all cases except SCG–650KC. The K_T_ parameter of the Temkin isotherm model, representing the binding constant, decreased with the increase in the temperature of adsorption in the cases of CHP adsorption onto SCG–650P, SCG–650C, and SCG–650KC. As previously stated, with the increase in the adsorption temperature, the binding of CHP molecules onto the materials was weaker. This finding was further confirmed with the E parameter from the Dubinin–Radushkevich isotherm, representing the mean free energy of adsorption; as in the mentioned cases, it also decreased. As the E values were way below 8 kJ mol^−1^, it can be concluded that the process was mainly physisorption on all the investigated materials at all temperatures.

#### 3.2.4. Thermodynamic Parameters

**Figure 6:**
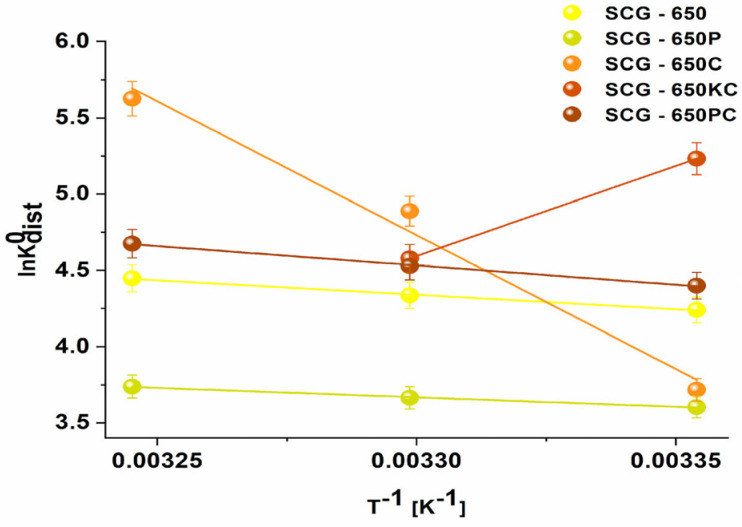
Van’t Hoff plots for CHP adsorption onto SCG–650 materials at 25, 30, and 35 °C.

Thermodynamics of the adsorption processes were also investigated. Figure 6 shows Van’t Hoff plots for CHP adsorption onto all materials, and Table 7 provides the obtained parameters with corresponding R^2^ values.

From Table 7, it can be concluded that the adsorption of CHP was, in all cases, spontaneous, as the values ΔG^0^ < 0. The CHP adsorption processes were endothermic with increasing entropy of the system, except for the adsorption onto SCG–650KC, as assumed previously. The process of CHP adsorption onto SCG–650C and SCG–650KC was highly dependent on the adsorption temperature. With the temperature increase, the efficiency of SCG–650C increased, while in the case of SCG–650KC, it decreased.

### 3.3. Regeneration and Reuse Under Environmentally Relevant Conditions

**Figure 7:**
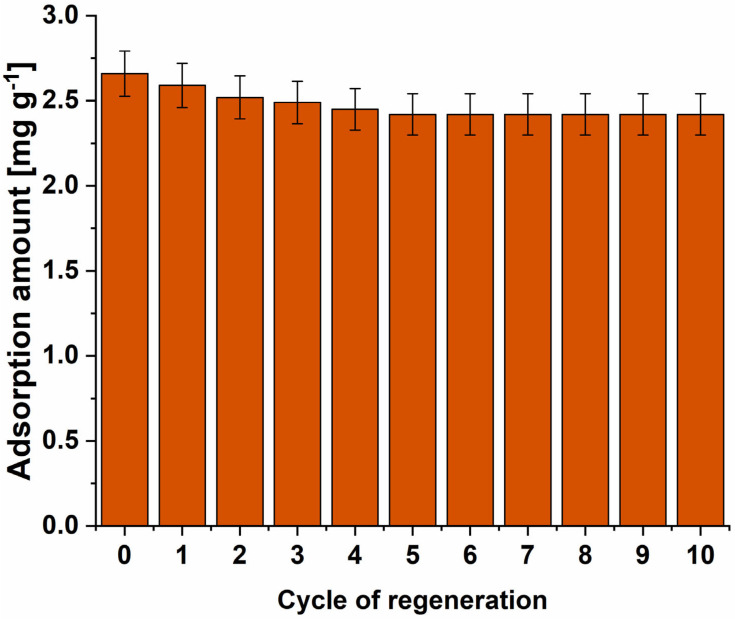
Regeneration and reuse of the material SCG–650KC at 25 °C.

The regeneration and reuse of SCG–650KC, the most promising material, were evaluated in tap water spiked with 5 × 10^−5^ mol dm^−3^ of CHP to simulate real-world conditions. The pH was maintained at 6, and the temperature was kept constant at 25 °C [33]. After adsorption, SCG–650KC was regenerated by washing with 5 mL of 96% ethanol, followed by separation and subsequent adsorption experiments over 10 cycles. The results, presented in Figure 7, demonstrate the material’s stability and performance.

Notably, the presence of ions in tap water did not affect CHP adsorption, as the results closely matched those obtained in deionized water. Over the first five cycles, a slight decrease in adsorption capacity was observed, but beyond that point, the material’s performance stabilized, suggesting that further regeneration and reuse are feasible without significant loss of efficiency.

### 3.4. Comparative Studies with Other Adsorbents

Few biomass-derived carbon materials have been used to remediate CHP from the environment. Various carbonization temperatures and activation processes have been used to synthesize carbon materials. Jacob et al. achieved an adsorption capacity for CHP removal of 3.20 mg g^−1^ using sugarcane bagasse merely carbonized at 450 °C [41]. Tobacco-derived carbon materials for this purpose were reported by Gonçalves et al. In their work, they tested the adsorption efficiency of carbonized tobacco at 750 °C and the effect of activating this material using ZnCl_2_ and NaOH. The adsorption capacities achieved in their work were 0.68, 1.60, and 0.46 mg g^−1^, respectively. This indicated that, as in this paper, chemical activation does not imply better efficiency [42]. Thuy et al. tested carbon materials obtained by carbonizing bamboo and coconut shells at 600 °C and subsequent activation using vapor steam for 2 h at 800–1000 °C. The obtained adsorption capacities were 0.59 mg g^−1^ for bamboo- and 0.50 mg g^−1^ for coconut shell-derived activated carbon material [43]. In their works, Katnić et al. investigated fig pomace- [44] and plum pomace- [45] derived materials, carbonized at 500 °C, and activated using gamma irradiation. Using fig pomace-derived material, the authors obtained a q_max_ value of 0.495 mg g^−1^, while using the plum pomace, the q_max_ value was 0.428 mg g^−1^ for CHP adsorption. An adsorption capacity of 12.37 mg g^−1^ was achieved in the work reported by Ettish et al. [46]. The biowaste material used in their work was cinnamon sticks, which were then subjected to the carbonization process at 900 °C and a physical activation using CO_2_. This work reports way higher adsorption capacities for CHP than the ones found in the literature for biowaste-activated carbon materials. The highest adsorption capacity was achieved for the material physically activated with CO_2_ for 1 h (51.7 ± 0.1 mg g^−1^). Comparing this result with the previous literature review, it can be concluded that SCG–650C adsorbed CHP more than four times more efficiently than the highest reported capacity (Ettish et al. [46]). 

## 4. Conclusions

This study highlights the development of biochar from SCG through carbonization at 650 °C and investigates the impact of various activation methods on the material’s adsorption performance. Under environmentally relevant conditions (25–35 °C, pH = 6, and realistic pesticide concentrations), the selective adsorption capabilities of SCG-derived biochars were tested with a focus on removing organophosphorus pesticides CHP and MLT. Chemically activated materials displayed a notably lower affinity for MLT, while materials activated with CO_2_ or a combined H_3_PO_4_/CO_2_ method showed enhanced adsorption for CHP, suggesting selective adsorption mechanisms based on surface charge and porosity. Among the tested materials, SCG–650KC demonstrated the highest adsorption capacity for CHP at lower temperatures, whereas SCG–650C exhibited a superior performance at elevated temperatures, with maximum adsorption capacities increasing from 2.5 to 51.7 mg·g^−1^ across the temperature range. The adsorption process was slow, reaching equilibrium after 24 h, and thermodynamic studies showed that higher temperatures generally improved CHP adsorption.

The primary advantage of SCG-derived biochar lies in its environmental impact rather than its economic efficiency. While these materials are regenerable, our focus is on their potential for sustainable waste valorization and environmental protection, transforming SCG waste into biochar that contributes to carbon capture by sequestering carbon and preventing organic decomposition. This approach aligns with broader environmental goals, supporting greenhouse gas reduction efforts and enhancing the ecological value of SCG biochars. Furthermore, these materials show potential applicability in the treatment of industrial pollutants, thus contributing to a circular economy and establishing the basis for sustainable solutions in environmental remediation.

## Figures and Tables

**Table 1:** Conditions for synthesis of the materials with corresponding yields.

Material	T_1_ [°C]	Y_1_ [%]	Grinding [min]	Chemical Activation	T_2_ [°C]	Physical Activation	Y_2_ [%]	Grinding [min]	Y [%] (Y_1_ × Y_2_)
SCG–650	650	18	15	/	/	/	/	/	18
SCG–650K	650	18	15	KOH	650	/	86	15	15.5
SCG–650P	650	18	15	H_3_PO_4_	650	/	86	15	15.5
SCG–650C	650	17	15	/	/	CO_2_	/	/	17
SCG–650KC	650	18	15	KOH	650	CO_2_	82	15	14.8
SCG–650PC	650	18	15	H_3_PO_4_	650	CO_2_	85	15	15.3

T_1_—temperature of carbonization; Y_1_—yield after carbonization; T_2_—temperature of activation; Y_1_—yield after activation; The symbol ‘/’ indicates that this step was not performed in the synthesis of the material.

**Table 2:** Chemical composition of the materials.

[at%]	C	O	N	K	P	Mg	Ca	Others *
SCG–650	82.1	8.7	8.1	0.6	0.1	0.2	0.2	0.0
SCG–650K	73.5	17.7	5.8	2.6	0.1	0.2	0.2	0.0
SCG–650P	79.0	12.2	6.3	0.7	1.2	0.2	0.3	0.0
SCG–650C	80.3	9.3	8.9	0.8	0.1	0.2	0.3	0.0
SCG–650KC	71.5	16.8	7.7	3.4	0.1	0.2	0.2	0.1
SCG–650PC	77.4	13.8	6.3	0.6	1.4	0.2	0.2	0.1

* Traces of Na, Cl, S, and Si.

**Table 3:** Specific surface areas by BET method (S_BET_) and total pore volumes (V_tot_) of the SCG–650 materials.

Sample	S_BET_ [m^2^ g^−1^]	V_tot_ × 10^3^ [cm^3^ g^−1^]
SCG–650	0.8	1.4
SCG–650K	2.2	4.1
SCG–650P	5.5	4.9
SCG–650C	1.3	0.5
SCG–650KC	98.0	44.4
SCG–650PC	0.7	0.8

**Table 4:** Screening test results (the percentage of adsorption of MLT and CHP with an initial concentration of 5 × 10^−5^ mol dm^−3^ on all the synthesized material within 1 h).

Sample	%_ads_ (MLT)	%_ads_ (CHP)
SCG–650	0.46 ± 0.02	9.5 ± 0.5
SCG–650K	0	0
SCG–650P	0	8.2 ± 0.4
SCG–650C	1.72 ± 0.09	11.0 ± 0.6
SCG–650KC	0	5.7 ± 0.3
SCG–650PC	1.98 ± 0.09	14.2 ± 0.7

**Table 5:** Kinetic parameters of CHP (5 × 10^−5^ mol dm^−3^) adsorption onto SCG–650 materials (1 mg·mL^−1^).

Material	SCG–650	SCG–650P	SCG–650C	SCG–650KC	SCG–650PC
Pseudo-first-order					
q_e_ [mg g^−1^]	3.3 ± 0.3	1.7 ± 0.7	1.94 ± 0.05	5.89 ± 0.02	3.70 ± 0.02
k_1_ × 10^2^ [min^−1^]	1.9 ± 0.4	1.2 ± 0.5	5.12 ± 0.06	0.324 ± 0.002	1.73 ± 0.02
χ^2^	0.394	0.153	0.028	0.120	0.053
R^2^	0.821	0.713	0.937	0.984	0.974
Pseudo-second-order					
q_e_ [mg g^−1^]	3.5 ± 0.5	1.9 ± 0.8	2.02 ± 0.03	7.33 ± 0.03	3.92 ± 0.02
k_2_ × 10^3^ [mg min^−1^ g^−1^]	6.1 ± 0.7	7.2 ± 0.8	43.1 ± 0.2	0.112 ± 0.002	6.24 ± 0.03
χ^2^	0.524	0.155	0.007	0.175	0.050
R^2^	0.763	0.709	0.984	0.976	0.975
Elovich model					
α × 10^1^ [mg g^−1^ min^−1^]	1.5 ± 0.9	0.40 ± 0.08	15.1 ± 0.8	0.262 ± 0.004	4.02 ± 0.04
β [g mg^−1^]	1.7 ± 0.7	2.7 ± 0.8	4.15 ± 0.08	0.487 ± 0.004	1.75 ± 0.05
χ^2^	0.793	0.183	0.033	0.298	0.092
R^2^	0.640	0.657	0.924	0.959	0.955
Intraparticle diffusion model					
I part					
C [mg g^−1^]	−2.39	−3.00	0.170	−2.430	−0.023
k_id_ [mg g^−1^ min^−0.5^]	0.669	0.549	0.210	0.444	0.302
R^2^	0.810	-	0.946	0.988	0.896
II part				-	
C [mg g^−1^]	3.041	1.104	1.619	−1.090	1.892
k_id_ [mg g^−1^ min^−0.5^]	0.006	0.018	0.012	0.271	0.078
R^2^	0.923	1.000	0.970	-	-
III part					
C [mg g^−1^]				2.532	3.019
k_id_ [mg g^−1^ min^−0.5^]				0.089	0.022
R^2^				0.986	0.999

**Table 6:** Parameters of CHP adsorption onto SCG–650 materials (1 mg ml^−1^) at 25, 30, and 35 °C.

Material	T (°C)	SCG–650	SCG–650P	SCG–650C	SCG–650KC	SCG–650PC
Freundlich isotherm						
K_F_ ×10^1^ [(mg g^−1^)(dm^3^ g^−1^)^1/n^]	25	1.89 ± 0.03	0.761 ± 0.002	1.32 ± 0.03	12 ± 2	4.2 ± 0.8
	30	3.28 ± 0.08	0.711 ± 0.007	2.47 ± 0.02	3.42 ± 0.05	4.5 ± 0.3
	35	4.7 ± 0.8	0.78 ± 0.07	3.48 ± 0.01	/	4.85 ± 0.05
n	25	1.40 ± 0.04	1.24 ± 0.06	1.49 ± 0.02	2.1 ± 0.2	1.8 ± 0.5
	30	1.67 ± 0.06	1.18 ± 0.05	1.21 ± 0.01	1.33 ± 0.06	1.8 ± 0.2
	35	1.8 ± 0.3	1.2 ± 0.2	1.07 ± 0.01	/	1.73 ± 0.04
Langmuir isotherm						
K_L_ ×10^2^ [dm^3^ mg^−1^]	25	2.81 ± 0.05	2.22 ± 0.03	3.53 ± 0.03	14.6 ± 0.6	7.9 ± 0.5
	30	6.52 ± 0.02	1.82 ± 0.03	1.61 ± 0.01	6.13 ± 0.05	7.74 ± 0.04
	35	8.8 ± 0.1	1.7 ± 0.1	0.624 ± 0.001	/	7.21 ± 0.01
q_max_ [mg g^−1^]	25	4.67 ± 0.04	2.98 ± 0.03	2.52 ± 0.02	7.14 ± 0.07	4.0 ± 0.2
	30	3.78 ± 0.03	3.53 ± 0.04	12.3 ± 0.1	5.42 ± 0.05	4.38 ± 0.03
	35	4.03 ± 0.09	3.94 ± 0.09	51.7 ± 0.1	/	4.96 ± 0.01
Temkin isotherm						
K_T_ × 10^1^ [dm^3^ mg^−1^]	25	6.67 ± 0.05	4.94 ± 0.02	7.25 ± 0.05	13.0 ± 0.4	7.9 ± 0.4
	30	7.60 ± 0.01	4.63 ± 0.03	6.02 ± 0.05	8.71 ± 0.01	8.62 ± 0.04
	35	8.57 ± 0.08	3.041 ± 0.001	5.97 ± 0.06	/	9.40 ± 0.02
b_T_ [J g mol^−1^mg^−1^]	25	3600 ± 40	5810 ± 30	6140 ± 40	1520 ± 60	2800 ± 100
	30	3190 ± 10	5440 ± 30	1950 ± 50	2490 ± 10	2710 ± 50
	35	2840 ± 70	4224 ± 5	1050 ± 60	/	2580 ± 30
Dubinin–Radushkevich isotherm						
q_DR_ [mg g^−1^]	25	1.9 ± 0.2	1.40 ± 0.03	1.2 ± 0.2	5.42 ± 0.03	2.78 ± 0.03
	30	2.4 ± 0.2	1.483 ± 0.001	3.55 ± 0.03	2.7 ± 0.2	2.9 ± 0.2
	35	2.86 ± 0.03	1.60 ± 0.01	9.34 ± 0.02	/	3.14 ± 0.06
E [J mol^−1^]	25	450 ± 30	148 ± 8	440 ± 30	686 ± 5	365 ± 7
	30	340 ± 30	562 ± 1	413 ± 4	480 ± 20	2200 ± 100
	35	418 ± 6	152 ± 2	144 ± 3	/	476 ± 8

The symbol ‘/’ indicates that data was not acquired.

**Table 7:** Thermodynamic parameters of CHP adsorption onto SCG–650 materials at 25, 30, and 35 °C.

	ΔH^0^ [kJ mol^−1^]	ΔS^0^ [J mol^−1^K^−1^]	ΔG^0^ [kJ mol^−1^]			R^2^
T (°C)			25	30	35	
SCG–650	15.8 ± 0.1	88.2 ± 0.1	−10.5 ± 0.1	−10.9 ± 0.1	−11.4 ± 0.1	0.994
SCG–650P	10.3 ± 0.1	64.5 ± 0.1	−8.92 ± 0.01	−9.23 ± 0.01	−9.62 ± 0.01	0.992
SCG–650C	146 ± 4	521 ± 5	−9.42 ± 0.04	−12.0 ± 0.2	−14.6 ± 0.3	0.971
SCG–650KC	−98.2	−285.7	−13.0	−11.5	−10.1	-
SCG–650PC	21.1 ± 0.1	107 ± 2	−10.9 ± 0.1	−11.4 ± 0.1	−12.0 ± 0.1	0.995

## Data Availability

The original contributions presented in the study are included in the article/supplementary material, further inquiries can be directed to the corresponding author.

## Supplementary Materials

The following supporting information can be downloaded at: https://www.mdpi.com/article/10.3390/ma17225499/s1, Figure S1: FTIR spectra of SCG–650C before and after the adsorption of CHP; Figure S2: Graphical representation of Temkin and Dubinin-Radushkevich isotherm models for CHP adsorption onto all investigated materials at 25, 30, and 35 °C; Table S1: Kinetic and isotherm models and their corresponding equations; Table S2: Parameteres of CHP adsorption onto materials (1 mg mL^−1^) at 25 °C; Table S3: Parameteres of CHP adsorption onto materials (1 mg mL^−1^) at 30 °C; Table S4: Parameteres of CHP adsorption onto materials (1 mg·mL^−1^) at 35 °C.
