# Supplementary material for: Transforming Food Biowaste into Selective and Reusable Adsorbents for Pesticide Removal from Water
Source: Materials (Basel). 2024 Nov 11;17(22):5499. doi: 10.3390/ma17225499 (PMC11595743; doi:10.3390/ma17225499)
Supplement: Supplementary file 1 [file materials-17-05499-s001.zip › materials-3299573-supplementary.pdf]

materials

MDPI

Supplementary Material

Transforming Food Biowaste into Selective and Reusable Adsorbents for Pesticide Removal from Water

Vedran Milanković [1,*], Tamara Tasić [1], Snežana Brković [1], Nebojša Potkonjak [1], Christoph Unterweger [2], Igor A. Pašti [3] and Tamara Lazarević-Pašti [1]

[1] Vinča Institute of Nuclear Sciences—National Institute of the Republic of Serbia, University of Belgrade, Mike Petrovica Alasa 12–14, 11000 Belgrade, Serbia; tamara.tasic@vin.bg.ac.rs (T.T.); snezana.mi-ulovic@vin.bg.ac.rs (S.B.); npotkonjak@vin.bg.ac.rs (N.P.); tamara@vin.bg.ac.rs (T.L.-P.)
[2] Wood K Plus—Kompetenzzentrum Holz GmbH, Altenberger Strasse 69, 4040 Linz, Austria; c.unterweger@wood-kplus.at
[3] Faculty of Physical Chemistry, University of Belgrade, Studentski Trg 12–16, 11158 Belgrade, Serbia; igor@ffh.bg.ac.rs
* Correspondence: vedran.milankovic@vin.bg.ac.rs

Table S1. Kinetic and isotherm models and their corresponding equations.

Pseudo-first order model	$q_t = q_e(1 - e^{-k_1 t})$	(S1)
Pseudo-second order model	$q_t = \frac{q_e^2 k_2 t}{1 + q_e k_2 t}$	(S2)
Elovich model	$q_t = \frac{1}{\beta}(1 + \alpha\beta t)$	(S3)
Intra-particle diffusion model	$q_t = k_{id} t^{0.5} + C$	(S4)
Freundlich isotherm	$q_t = K_F C_e^{\frac{1}{n}}$	(S5)
Langmuir isotherm	$q_t = \frac{q_{max} K_L C_e}{1 + K_L C_e}$	(S6)
Temkin isotherm	$q_t = \frac{RT}{b_T} ln K_T C_e$	(S7)
Dubinin – Radushkevich isotherm	$q_t = q_{DR} e^{-K_{DR}\varepsilon^2}$	(S8)

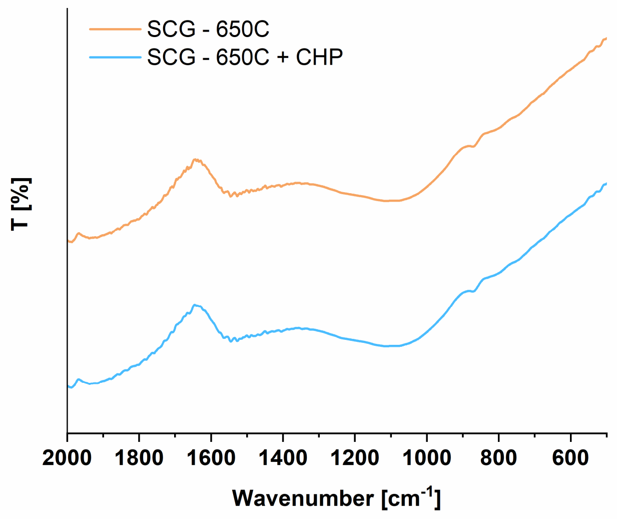

Figure S1. FTIR spectra of SCG – 650C before and after the adsorption of CHP.

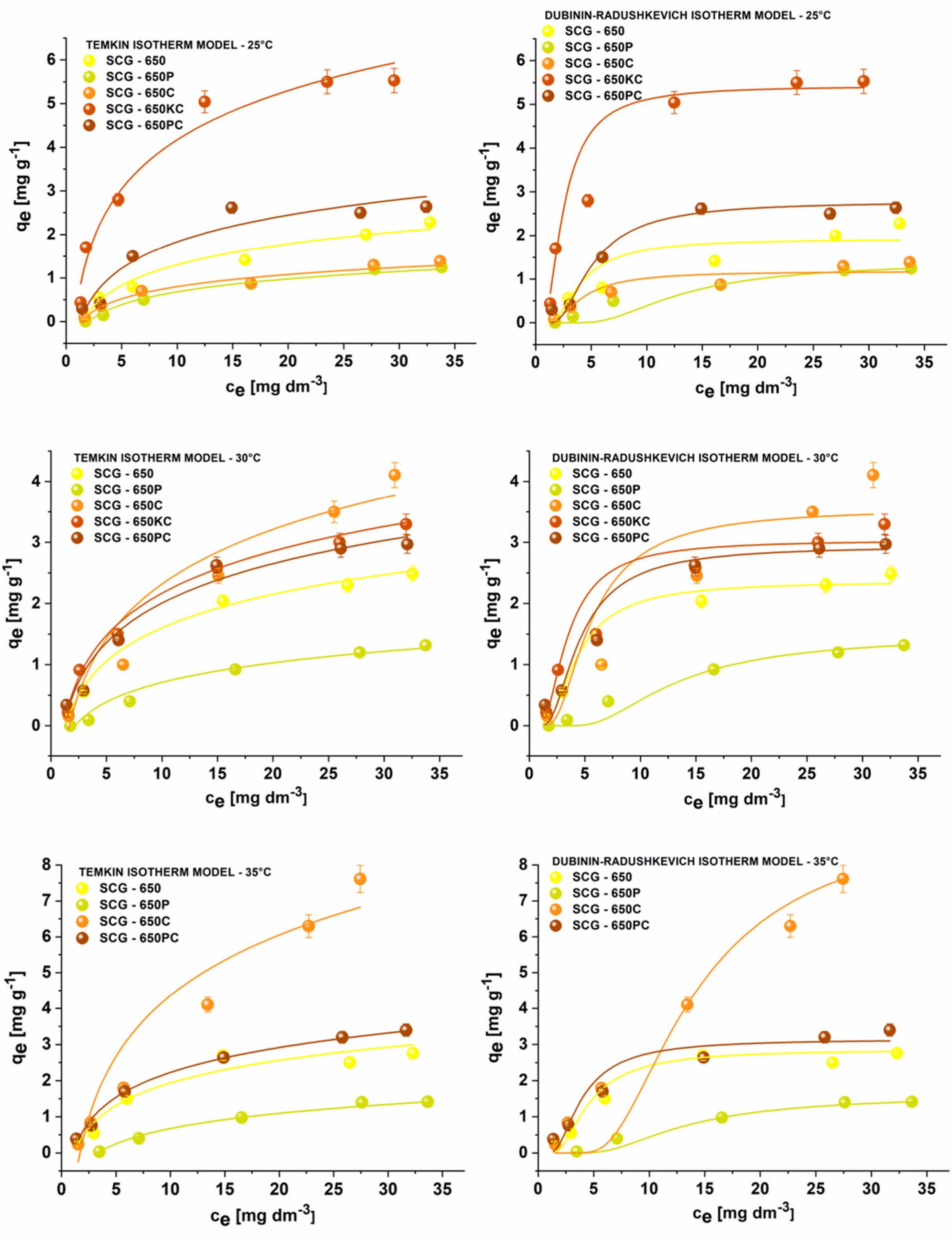

Figure S2. Graphical representation of Temkin and Dubinin-Radushkevich isotherm models for CHP adsorption onto all investigated materials at 25, 30, and 35 °C.

Table S2. Parameteres of CHP adsorption onto materials (1 mg mL^{-1}) at 25 °C.

Material	SCG – 650	SCG – 650P	SCG – 650C	SCG – 650KC	SCG – 650PC
**Freundlich isotherm**					
K_F ×10^1 [(mg g^{-1})(dm^3 g^{-1})$^{1/n}$]	1.89 ± 0.03	0.761 ± 0.002	1.32 ± 0.03	12 ± 2	4.2 ± 0.8
n	1.40 ± 0.04	1.24 ± 0.06	1.49 ± 0.02	2.1 ± 0.2	1.8 ± 0.5
χ^2	0.035	0.019	0.008	1.109	0.391
R^2	0.963	0.945	0.975	0.822	0.772
**Langmuir isotherm**					
KL ×10^2 [dm^3 mg^{-1}]	2.81 ± 0.05	2.22 ± 0.03	3.53 ± 0.03	14.6 ± 006	7.9 ± 0.5
q_{max} [mg g^{-1}]	4.67 ± 0.04	2.98 ± 0.03	2.52 ± 0.02	7.14 ± 0.07	4.0 ± 0.2
χ^2	0.034	0.010	0.009	0.406	0.204
R^2	0.965	0.970	0.972	0.935	0.881
**Temkin isotherm**					
K_t × 10^1 [dm^3 mg^{-1}]	6.67 ± 0.05	4.94 ± 0.02	7.25 ± 0.05	13.0 ± 0.4	7.9 ± 0.4
b_t [J g mol^{-1} mg^{-1}]	3600 ± 40	5810 ± 30	6140 ± 40	1520 ± 60	2800 ± 100
χ^2	0.042	0.006	0.014	0.357	0.204
R^2	0.956	0.984	0.959	0.943	0.881
**Dubinin-Radushkevich isotherm**					
K_{dr} ×10^6 [mol^2 J^{-2}]	2.5 ± 0.3	23.2 ± 0.4	2.6 ± 0.3	1.06 ± 0.03	3.75 ± 0.05
q_{dr} [mg g^{-1}]	1.9 ± 0.2	1.40 ± 0.03	1.2 ± 0.2	5.42 ± 0.03	2.78 ± 0.03
E [J mol^{-1}]	450 ± 30	148 ± 8	440 ± 30	686 ± 5	365 ± 7
χ^2	0.156	0.011	0.055	0.070	0.051
R^2	0.835	0.969	0.832	0.989	0.970

Table S3. Parameteres of CHP adsorption onto materials (1 mg·mL^{-1}) at 30 °C.

Material	SCG – 650	SCG – 650P	SCG – 650C	SCG – 650KC	SCG – 650PC
**Freundlich isotherm**					
K_F ×10^1 [(mg g^{-1})(dm^3 g^{-1})$^{1/n}$]	3.28 ± 0.08	0.711 ± 0.007	2.47 ± 0.02	3.42 ± 0.05	4.5 ± 0.3
n	1.67 ± 0.06	1.18 ± 0.05	1.21 ± 0.01	1.33 ± 0.06	1.8 ± 0.2
χ^2	0.104	0.027	0.033	0.109	0.232
R^2	0.917	0.934	0.990	0.926	0.875
**Langmuir isotherm**					
K_L ×10^2 [dm^3 mg^{-1}]	6.52 ± 0.02	1.82 ± 0.03	1.61 ± 0.01	6.13 ± 0.05	7.74 ± 0.04
q_{max} [mg g^{-1}]	3.78 ± 0.03	3.53 ± 0.04	12.3 ± 0.1	5.42 ± 0.05	4.38 ± 0.03
χ^2	0.028	0.018	0.012	0.088	0.082
R^2	0.977	0.957	0.996	0.940	0.956
**Temkin isotherm**					
K_t×10^1 [dm^3 mg^{-1}]	7.60 ± 0.01	4.63 ± 0.03	6.02 ± 0.05	8.71 ± 0.01	8.62 ± 0.04
b_t [J g mol^{-1}mg^{-1}]	3190 ± 10	5440 ± 30	1950 ± 50	2490 ± 10	2710 ± 50
χ^2	0.010	0.013	0.167	0.013	0.092
R^2	0.992	0.969	0.950	0.991	0.951
**Dubinin-Radushkevich isotherm**					
K_{dr} ×10^6 [mol^2 J^{-2}]	4.2 ± 0.2	1.582 ± 0.001	2.93 ± 0.02	2.2 ± 0.2	0.11 ± 0.02
q_{dr} [mg g^{-1}]	2.4 ± 0.2	1.483 ± 0.001	3.55 ± 0.03	2.7 ± 0.2	2.9 ± 0.2
E [J mol^{-1}]	340 ± 30	562 ± 1	413 ± 4	480 ± 20	2200 ± 100
χ^2	0.516	0.000	0.061	1.842	7.930

R^2	0.844	1.000	0.967	0.869	0.866

Table S4. Parameteres of CHP adsorption onto materials (1 mg mL^{-1}) at 35 °C.

Material	SCG – 650	SCG – 650P	SCG – 650C	SCG – 650KC	SCG – 650PC
**Freundlich isotherm**					
K_F ×10^1 [(mg g^{-1})(dm^3 g^{-1})$^{1/n}$]	4.7 ± 0.8	0.78 ± 0.07	3.48 ± 0.01	/	4.85 ± 0.05
n	1.8 ± 0.3	1.2 ± 0.2	1.07 ± 0.01	/	1.73 ± 0.04
χ^2	0.358	0.065	0.055	/	0.095
R^2	0.798	0.869	0.995	/	0.955
**Langmuir isotherm**					
K_L × 10^2 [dm^3 mg^{-1}]	8.8 ± 0.1	1.7 ± 0.1	0.624 ± 0.001	/	7.21 ± 0.01
q_{max} [mg g^{-1}]	4.03 ± 0.09	3.94 ± 0.09	51.7 ± 0.1	/	4.96 ± 0.01
χ^2	0.162	0.047	0.035	/	0.007
R^2	0.908	0.905	0.997	/	0.997
**Temkin isotherm**					
K_t × 10^1 [dm^3 mg^{-1}]	8.57 ± 0.08	3.041 ± 0.001	5.97 ± 0.06	/	9.40 ± 0.02
b_t [J g mol^{-1}mg^{-1}]	2840 ± 70	4224 ± 5	1050 ± 60	/	2580 ± 30
χ^2	0.161	0.000	0.945	/	0.026
R^2	0.909	1.000	0.918	/	0.988
**Dubinin-Radushkevich isotherm**					
K_{dr} × 10^6 [mol^2 J^{-2}]	2.87 ± 0.03	21.7 ± 0.1	24.2 ± 0.3	/	2.21 ± 0.06
q_{dr} [mg g^{-1}]	2.86 ± 0.03	1.60 ± 0.01	9.34 ± 0.02	/	3.14 ± 0.06
E [J mol^{-1}]	418 ± 6	152 ± 2	144 ± 3	/	476 ± 8
χ^2	0.048	0.001	0.380	/	0.155
R^2	0.973	0.998	0.967	/	0.927
